# Impact of smoking on urologic cancers: a snapshot of current evidence

**DOI:** 10.1007/s00345-023-04406-y

**Published:** 2023-04-24

**Authors:** Raj Kumar, Richard Matulewicz, Andrea Mari, Marco Moschini, Saum Ghodoussipour, Benjamin Pradere, Michael Rink, Riccardo Autorino, Mihir M. Desai, Inderbir Gill, Giovanni E. Cacciamani

**Affiliations:** 1 The Catherine and Joseph Aresty Department of Urology, USC Institute of Urology, Keck School of Medicine, University of Southern California, Los Angeles, USA; 2 Department of Surgery, Urology Service, Memorial Sloan Kettering Cancer Center, New York, NY USA; 3 Department of Urology, Careggi Hospital, University of Florence, Florence, Italy; 4 Department of Urology, La Croix du Sud Hospital, 31130 Quint Fonsegrives, France; 5 Bladder and Urothelial Cancer Program, Rutgers Cancer Institute of New Jersey, New Brunswick, NJ USA; 6 Department of Urology, Comprehensive Cancer Center, Medical University of Vienna, Vienna, Austria; 7 Department of Urology, University Medical Center Hamburg-Eppendorf, Hamburg, Germany; 8 Division of Urology, Department of Surgery, Virginia Commonwealth University, Richmond, VA USA

**Keywords:** Smoking, Urologic oncology, Tumor recurrence, Smoking cessation

## Abstract

**Purpose:**

The purpose of this paper is to present evidence regarding the associations between smoking and the following urologic cancers: prostate, bladder, renal, and upper tract urothelial cancer (UTUC).

**Methods:**

This is a narrative review. PubMed was queried for evidence-based analyses and trials regarding the associations between smoking and prostate, bladder, renal, and UTUC tumors from inception to September 1, 2022. Emphasis was placed on articles referenced in national guidelines and protocols.

**Results:**

Prostate—multiple studies associate smoking with higher Gleason score, higher tumor stage, and extracapsular invasion. Though smoking has not yet been linked to tumorigenesis, there is evidence that it plays a role in biochemical recurrence and cancer-specific mortality. Bladder—smoking is strongly associated with bladder cancer, likely due to DNA damage from the release of carcinogenic compounds. Additionally, smoking has been linked to increased cancer-specific mortality and higher risk of tumor recurrence. Renal—smoking tobacco has been associated with tumorigenesis, higher tumor grade and stage, poorer mortality rates, and a greater risk of tumor recurrence. UTUC—tumorigenesis has been associated with smoking tobacco. Additionally, more advanced disease, higher stage, lymph node metastases, poorer survival outcomes, and tumor recurrence have been linked to smoking.

**Conclusion:**

Smoking has been shown to significantly affect most urologic cancers and has been associated with more aggressive disease, poorer outcomes, and tumor recurrence. The role of smoking cessation is still unclear, but appears to provide some protective effect. Urologists have an opportunity to engage in primary prevention by encouraging cessation practices.

## Introduction

**Fig. 1:**
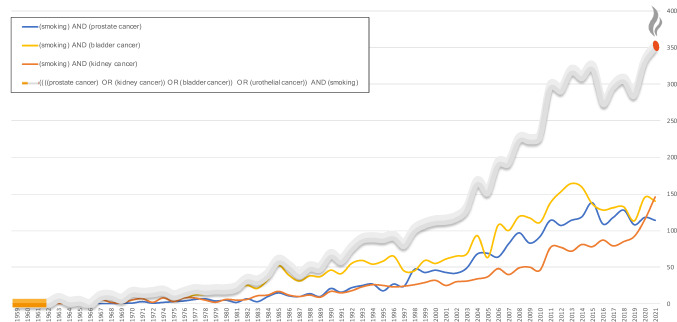
Trends over time in the number of publications associating smoking with urologic cancers. (Search in PubMed September 1, 2022)

The association between smoking tobacco and urologic cancers has been extensively studied. While it is well known that smoking is among the leading predisposing factors for bladder cancer, prostate cancer, renal cancer, and upper tract urothelial cancer (UTUC) [1–4], the impact of smoking tobacco can be manifold as it may affect several stages in cancer therapy. In addition to tumorigenesis, smoking may also lead to poor surgical outcomes, prolonged recovery periods, increased complication rate, poor response to chemotherapy, and potentially have an impact on recurrence [5–8]. Studies and publications regarding urologic cancers and smoking have gradually been on the rise over the last fifty years (Fig. 1).

It should be noted that—just as cessation methods are evolving—consumption methods are evolving as well. Tobacco is more frequently partaken through the use of e-cigarettes and waterpipes (also known as hookah or shisha). While data on these methods is lacking, preliminary studies reveal potential evidence of cytotoxic effects, cell damage, DNA damage, and oxidative stress [9–11].

Importantly, cancer and subsequent therapy is a powerful teaching moment for patients. The role of the urologist in smoking cessation should not be understated, and efforts to quit tobacco and its products should be undertaken if possible. Evidence has demonstrated that cessation efforts by urologists can have significant effects on smoking cessation and readiness to quit [12]. Furthermore—unlike with lung cancer—patients are often unaware of the strong evidence linking smoking to urologic cancers [13]. Urologists should be aware of methods to counsel patients on smoking cessation, including assessment of patient motivation and evidence-based therapies [14].

In this narrative review, we observe and compile current evidence regarding the impact of smoking on the following cancers: prostate, bladder, renal, and UTUC.

## Prostate cancer

### Etiology

Prostate cancer (PCa) is the second most diagnosed cancer in men worldwide [15]. Many potential risk factors for prostate cancer have been studied. Current smoking status has not explicitly been linked with tumorigenesis. The most recent report by the Surgeon General in 2014 concluded that “the evidence is suggestive of no causal relationship between smoking and the risk of incident prostate cancer [16].”

However, while the existence and mechanism of an etiologic link between smoking and prostate cancer is unclear, there have been several proposed mechanisms. The prevalent hypothesis involves the mutation of various cancer progression genes by tobacco-related carcinogens. Other proposals involve inflammatory response, hormone alterations, proliferation of tumor angiogenesis, and immune suppression [17, 18].

### Extent of disease

The same report by the Surgeon General in 2014 also concluded that “in men who have prostate cancer, the evidence is suggestive of a higher risk of advanced-stage disease and less-well-differentiated cancer in smokers than in non-smokers, and—independent of stage and histologic grade—a higher risk of disease progression” [16].

A study by Mari et al. in 2017 assessed 214 patients, and found that patients with a higher cumulating smoking history reported a higher pathologic Gleason score (*p* = 0.01) and extracapsular extension (*p* = 0.004) [19].

Another study by Kenfield et al. included 5366 patients. This study found that 14.7% of patient with current smoking status had stage T3 or higher disease compared to 8.3% of never-smokers. Additionally, 16.0% of current smokers had a Gleason score of 7 or more compared to 10.7% of never-smokers [20].

### Complications

Smoking is a known risk factor for poorer surgical recovery times and increased post-operative complications [8]. Additionally, smoking has been associated with a higher risk of prostate cancer mortality. A large, longitudinal prospective study by Kenfield et al. observed that—compared with men who never smoked—current smoking status was associated with a 60% higher risk of prostate cancer mortality [20]. This study also showed that smoking cessation for a period of 10 years or more prior to diagnosis had a similar mortality and recurrence rate to that of never-smokers.

### Tumor recurrence

Tobacco smoking has been associated with recurrence of disease following therapy for prostate cancer.

A systematic review and meta-analysis by Foerster et al. analyzed a total of 16 studies assessing the effect of smoking tobacco on biochemical recurrence. These studies observed 21,797 participants over a median of 61 months. Of the studies assessing the effects of current smoking status, 10 studies had a hazard ratio permitting inclusion into the meta-analysis, which showed a significant association with biochemical recurrence (HR = 1.40, *p* < 0.001). Similarly, an integration of 7 studies showed that former smoking status was significantly associated with biochemical recurrence (HR = 1.19, *p* < 0.001) [21].

One retrospective study that specifically analyzed the effects of smoking status on risk of biochemical recurrence found that current and former smoking status was significantly associated with higher risk of biochemical recurrence. Additionally, it was found that smoking cessation for 10 years or more conferred the same risk of biochemical recurrence as a never-smoking status. This same study found that cumulative exposure was not significantly associated with biochemical recurrence [22].

Another study by Kenfield et al. conveyed similar findings—that smoking cessation for 10 years or more conferred a biochemical recurrence risk comparable to that of never-smokers. However, this study also found that former smokers with less than 10 years of cessation and with less than 20 pack-years of smoking had a cancer mortality risk comparable to never-smokers as well [20].

## Bladder cancer

### Etiology

In 2022, urothelial carcinoma of the bladder is estimated to cause 81,180 newly diagnosed cancers and 17,100 deaths in the United States [23]. Tobacco smoking is a well-known and established cause of bladder cancer [16], and has been linked to nearly half of all cases of bladder cancer and has been associated with poor outcomes following diagnosis [1, 24, 25].

The risk of bladder cancer is estimated to be between two to four times higher for current smokers and three times higher for former smokers [24]. The etiology of tumorigenesis with regard to smoking is unclear. However, it is well known that tobacco is a strong source of carcinogens, such as polycyclic aromatic hydrocarbons, aromatic amines, heterocyclic amines, and N-nitroso compounds. These compounds are known to cause DNA damage, and have been strongly associated with bladder cancer [26]. Proposed tumorigenic mechanisms including mutation of the tumor suppressor gene TP53 [27] and mutations within chromosome 9 [28].

### Extent of disease

It is unclear if smoking is associated with higher grade or stage of bladder cancer at diagnosis. Several studies show a potential association, with several possible acknowledged confounding factors. One study by Chamssudin et al. evaluated 300 patients with bladder cancer to assess if smoking status could be associated with advance tumor grade or stage. While the study could not find a significant difference between tumor grade (*p* = 0.702) or stage (*p* = 0.166) between smokers and non-smokers, high-dose smokers were found to have significantly higher grade (*p* = 0.026) and stage (*p* = 0.037) compared to other assessed groups [29].

A retrospective study by Pietzak et al. queried the records of 1795 patients with urothelial cell carcinoma of the bladder. A multivariate analysis showed that patients who met the National Lung Cancer Screening Trial selection criteria were at a higher risk of initial muscle invasive tumor (OR = 2.5, *p* < 0.001) and were at increased risk of a higher grade initial tumor (OR = 2.76, *p* = 0.004) [30].

A retrospective study by Barbosa et al. in 2018 assessed 1859 patients. Among former smokers, increased smoking amount per day (*p* = 0.008), duration in years (*p* = 0.007), and cumulative amount in pack-years (*p* = 0.003) was associated with increased tumor aggressiveness. However, this association was not observed in current smokers [31].

Finally, a pooled analysis of 11 case–control studies by Brennan et al. showed a gradual reduction in the risk of bladder cancer following smoking cessation. This reduction is described as near 40% decreased risk following 1–4 years of cessation and progresses up to nearly 60% following 25 years of cessation. However, the risk was reported never to fall to the same risk as that of never-smokers [32].

### Complications

Compared to non-smokers, smoking has been linked to poor peri-operative outcomes following therapy. Mortality, infection rate, and complication rate are all found to be increased in patients with current smoking status [33, 34].

A systematic review and meta-analysis by Tellini et al. analyzed 11 studies, 10 of which assessed the impact of smoking status on major postoperative complications. Smoking status was found to be significantly associated with major postoperative complications (HR = 1.87, 95% CI 1.51–2.32) [33].

A large-scale database analysis by Al Hussein Al Awamlh et al. assessed 493,282 patients, 42.6% of whom reported having smoked tobacco. This study showed a significant association between smoking initiation in teenage years and bladder cancer-specific mortality (HR = 2.14, 95% CI 1.28–3.56) [35].

Smoking tobacco has been associated with poorer outcomes and recovery following both trans-urethral resection and radical cystectomy [7, 24]. One systematic review by Rink et al. found that 10 out of 16 analyzed studies associated a current smoking status with significant risk of disease recurrence [24]. Furthermore, smoking has been associated with decreased efficacy of intravesical chemotherapy and immunotherapy [24]. Finally, smoking has been associated with poorer recovery times and post-operative complications following surgical procedures [8].

The role of smoking cessation should not be understated. For cases of non-muscle invasive bladder cancer, smoking cessation has been shown to decrease the risk of disease progression and progression [36]. Though smoking has been associated with a poorer prognosis for radical cystectomy following bladder cancer, Rink et al. found in 2013 that the association was dose-dependent and mitigated by smoking cessation for more than 10 years [37].

### Recurrence

Smoking status has been associated with recurrence of bladder cancer. One systematic review and meta-analysis found that current smokers have a significantly higher risk for recurrence than former and never-smokers (Hazard Ratio (HR) = 1.24, *p* < 0.0001). In an analysis of patients who specifically underwent neoadjuvant chemotherapy, smokers were found to have a higher rate of recurrence (HR = 1.35, *p* = 0.08), but this was not statistically significant [38].

Rink et al. in 2012 found a dose–response relationship between smoking and disease recurrence. Heavy short-term smoking (HR = 1.54), light long-term (HR = 1.70), and heavy long-term smoking (HR = 2.22) were all found to be associated with a progressively higher, statistically significant risk of disease recurrence compared to light short-term smoking. Additionally, smoking cessation for 10 years or more was associated with a lower risk of disease recurrence (HR = 0.44) [37].

Wang et al. found a similar association, where light long-term (HR = 1.4), heavy short-term (HR = 1.4), and heavy long-term smoking (HR = 2.0) all conveyed a higher risk of disease recurrence compared to light short-term smoking [39].

## Renal cancer

### Etiology

The strong association between smoking and renal cancer has been known for decades, and even published in the 1982 report of the surgeon general [40], which stated in no uncertain terms: “Cigarette smoking is a contributory factor in the development of kidney cancer in the United States.” Modern data has only strengthened this association. Similar to bladder cancer, renal cancer has been associated with the carcinogenic compounds, such as polycyclic aromatic hydrocarbons and aromatic amines, released by tobacco smoking [26].

### Extent of disease

In particular, smoking and increased smoking intensity have been linked to renal cell carcinoma (RCC) [41, 42], though it has also been associated with chromophobe carcinoma and collecting duct/medullary carcinoma [43].

A Study by Macleod et al. found a dose-dependent increase in risk for RCC. Patients who had smoked up to 7.5 pack-years experienced a slightly increased risk of RCC (HR = 1.15, 95% CI 0.77–1.71). This rate increased for patient who smoked between 22.5 and 37.5 pack-years (HR = 1.67, 95% CI 1.16–2.42) [41].

An analysis by Lotan et al. utilized the data from the Prostate, Lung, Colorectal, and Ovarian (PLCO) Cancer Screening Trial and the National Lung Screening Trial (NLST). The PLCO data generally showed a gradually increasing trend in RCC incidence as cumulative smoking in pack-years increased up to ≥ 50 pack-years (HR = 2.06, 95% CI 1.52–2.78). Similarly, the NLST showed a dose-dependent increase in RCC risk with cumulative smoking trends up to ≥ 50 pack-years (HR = 1.43, 95% CI 0.95–2.14) [42].

A study of 30,282 RCC cases by Gansler et al. in 2020 showed an increase in an adjusted prevalence ratio (aPR) for chromophobe RCC in smokers (aPR = 0.88, 95% CI 0.81–0.95) compared to non-smokers (aPR = 0.58, 95% CI 0.50–0.67) [43].

One meta-analysis by Cumberbatch et al. found the relative risk of developing RCC was significantly higher for smokers (RR = 1.31, *p* < 0.001) when compared to non-smokers. This risk was increased in current smokers (RR = 1.36, *p* < 0.001) [44].

A study by Setiawan et al. analyzed 347 cases of RCC, found an increase in incidence in RCC in men (RR = 2.34, 95% CI 1.43–3.82) and women (RR = 1.63, 95% CI 0.88–3.00). This risk decreased with former smoking status in men (RR = 1.46, 95% CI 1.02–2.09) and (RR = 1.30, 95% CI 0.85–1.99) in women [45].

Smoking has been associated with higher stage cancer and more aggressive phenotypes at diagnosis. This in turn likely increases disease-specific mortality. A study by Sweeney et al. assessed 132 cases of RCC, finding that current smokers were slightly over twice as likely to be diagnosed with distant or unknown stage disease compared to non-smokers (OR = 2.2, 95% CI 1.4–3.5) [46].

### Complications

Smoking has been linked to poor perioperative outcomes, recovery, post-operative complications, and disease progression. Additionally, smoking has been linked to poorer disease-specific mortality. The aforementioned study by Sweeney et al. found that current smokers experienced an increased risk of death following RCC diagnosis (HR = 2.5, 95% CI 1.5–4.3) compared to former smokers (HR = 1.2, 95% CI 0.7–2.1) [46].

### Recurrence

Data regarding any association between smoking tobacco and recurrence of renal cancer is lacking, but in 2019, van der Mjin et al. analyzed 873 patients and found that a history of smoking was not significantly associated with recurrence of RCC (HR = 1.159, *p* = 0.430). Rather, other risk factors were found to be significantly associated, including male gender, increased T stage, nuclear grade, clear cell histology, and presence of diabetes or hypertension.

## Upper tract urothelial carcinoma

### Etiology

Similar to bladder cancer and renal cancer, UTUC has been associated with tobacco smoking and the carcinogenic compounds released upon smoking [47]. UTUC is often treated with radical nephroureterectomy (RNU) and follow-up involves careful monitoring for intra-vesicular tumor recurrence or other disease recurrence.

### Extent of disease

In 2013, Rink et al. associated current smoking status with higher risk of advanced disease status and lymph node metastases [47]. This study also found a dose-dependent association between tobacco smoking and advanced disease as well as poor surgical outcomes. Smoking cessation for more than 10 years was associated with less advanced disease stages, fewer disease recurrences, and lower cancer-specific mortality compared with recent former or current smokers [47].

Another study by Rink et al. assessed 865 patients undergoing RNU for UTUC, and found that current smokers had a higher stage (*p* ≤ 0.037) and more lymph node metastases (*p* ≤ 0.003) compared to non-smokers [48].

One study by Miyata et al. in 2014 retrospectively analyzed 134 cases of non-metastatic UTUC. This study found no significant difference in clinicopathological characteristics of tumors based on smoking status [49].

### Complications

Smoking tobacco has been associated with fewer post-surgical complications [8]. Cigarette smoking has also been associated with a shorter disease-specific survival and is a poor prognostic indicator [50]. However, the impact size of this association is unclear, and there may be associated confounding variables.

In the aforementioned Rink et al. study assessing 865 patients, more current smokers were treated with open surgery rather than minimally invasive procedures (p ≤ 0.047), which may cause increased and higher grade complications [48].

### Recurrence

Following radical nephroureterectomy for UTUC, studies have shown that smoking has been associated with intravesical recurrence, disease recurrence, and cancer-specific mortality.

One systematic review found that the majority of studies following patients after radical nephroureterectomy for UTUC were able to associate tobacco smoking with intravesical recurrence, disease recurrence, cancer-specific mortality, and/or any cause mortality [36]. This review was unable to find evidence that smoking cessation affects recurrence of UTUC following therapy.

A study in 2012 by Rink et al. found that current smokers had a significantly increased risk of UTUC disease recurrence after therapy. In addition, both current and former smokers had a higher risk of cancer-specific mortality. Additionally, a dose-dependent relationship was found between smoking and the risk of disease recurrence. Heavy-long-term smoking was found to be independently associated with the highest risk of disease recurrence [47].

Another study by Rink et al. containing 865 patients found that in men, current smokers experienced an increased risk of recurrence (*p* ≤ 0.031). In women, a similar risk of recurrence was observed (*p* ≤ 0.003).

Additionally, the aforementioned paper by Miyata et al. analyzed 134 patients, and found that the smoking status of a patient was a significant predictor of tumor recurrence (*p* = 0.045) and metastases (log-rank *p* = 0.016).

A meta-analysis by Crivelli et al. in 2014 found that of six criteria-appropriate studies that assessed intra-vesicular recurrence, five found a statistically significant increase in risk associated with smoking tobacco [36].

## Conclusion

Smoking has been firmly linked to most urologic cancers including prostate, bladder, renal, and upper tract urothelial cancers. In general, tobacco smoking has been associated with higher grade and stage tumors, increased disease recurrence, poor surgical outcomes, worse postoperative complications, disease progression, and tumor recurrence. Smoking cessation appears to have beneficial effects with most urologic cancers, particularly if maintained in the long term. Curbing smoking habits is therefore critical for urologic health and should be a priority for urologists.

## Data Availability

Not applicable.

## References

[1] Mori K, Mostafaei H, Abufaraj M, Yang L, Egawa S, Shariat SF (2020). Smoking and bladder cancer: review of the recent literature. Curr Opin Urol.

[2] Miyazaki J, Nishiyama H (2017). Epidemiology of urothelial carcinoma. Int J Urol.

[3] Pernar CH, Ebot EM, Wilson KM, Mucci LA (2018). The epidemiology of prostate cancer. Cold Spring Harb Perspect Med..

[4] Capitanio U, Bensalah K, Bex A (2019). Epidemiology of renal cell carcinoma. Eur Urol.

[5] Hagiwara M, Kikuchi E, Tanaka N (2013). Impact of smoking status on bladder tumor recurrence after radical nephroureterectomy for upper tract urothelial carcinoma. J Urol.

[6] Li HM, Azhati B, Rexiati M (2017). Impact of smoking status and cumulative smoking exposure on tumor recurrence of non-muscle-invasive bladder cancer. Int Urol Nephrol.

[7] Cacciamani GE, Matulewicz RS, Kumar R (2021). Fighting the 'tobacco epidemic'—a call to action to identify Targeted Intervention Points (TIPs) for better counseling patients with urothelial cancer. Urol Oncol.

[8] Gronkjaer M, Eliasen M, Skov-Ettrup LS (2014). Preoperative smoking status and postoperative complications: a systematic review and meta-analysis. Ann Surg.

[9] Ramoa CP, Eissenberg T, Sahingur SE (2017). Increasing popularity of waterpipe tobacco smoking and electronic cigarette use: implications for oral healthcare. J Periodontal Res.

[10] Sundar IK, Javed F, Romanos GE, Rahman I (2016). E-cigarettes and flavorings induce inflammatory and pro-senescence responses in oral epithelial cells and periodontal fibroblasts. Oncotarget.

[11] Yu V, Rahimy M, Korrapati A (2016). Electronic cigarettes induce DNA strand breaks and cell death independently of nicotine in cell lines. Oral Oncol.

[12] Bjurlin MA, Cohn MR, Kim DY (2013). Brief smoking cessation intervention: a prospective trial in the urology setting. J Urol.

[13] Sosnowski R, Przewozniak K (2015). The role of the urologist in smoking cessation: why is it important?. Urol Oncol.

[14] Affentranger A, Matulewicz RS, Fankhauser CD (2022). Why and how smoking cessation must be implemented in urology clinics as a standard of care. Eur Urol.

[15] Culp MB, Soerjomataram I, Efstathiou JA, Bray F, Jemal A (2020). Recent global patterns in prostate cancer incidence and mortality rates. Eur Urol.

[16] In: The Health Consequences of Smoking-50 Years of Progress: A Report of the Surgeon General. Atlanta (GA)2014.

[17] Zu K, Giovannucci E (2009). Smoking and aggressive prostate cancer: a review of the epidemiologic evidence. Cancer Causes Control.

[18] Moreira DM, Nickel JC, Gerber L (2015). Smoking is associated with acute and chronic prostatic inflammation: results from the REDUCE study. Cancer Prev Res (Phila).

[19] Mari A, Abufaraj M, Foerster B (2018). Oncologic effect of cumulative smoking exposure in patients treated with salvage radical prostatectomy for radiation-recurrent prostate cancer. Clin Genitourin Cancer.

[20] Kenfield SA, Stampfer MJ, Chan JM, Giovannucci E (2011). Smoking and prostate cancer survival and recurrence. JAMA.

[21] Foerster B, Pozo C, Abufaraj M (2018). Association of smoking status with recurrence, metastasis, and mortality among patients with localized prostate cancer undergoing prostatectomy or radiotherapy: a systematic review and meta-analysis. JAMA Oncol.

[22] Rieken M, Shariat SF, Kluth LA (2015). Association of cigarette smoking and smoking cessation with biochemical recurrence of prostate cancer in patients treated with radical prostatectomy. Eur Urol.

[23] Siegel RL, Miller KD, Fuchs HE, Jemal A (2022). Cancer statistics, 2022. CA Cancer J Clin.

[24] Rink M, Crivelli JJ, Shariat SF, Chun FK, Messing EM, Soloway MS (2015). Smoking and bladder cancer: a systematic review of risk and outcomes. Eur Urol Focus.

[25] van Osch FH, Jochems SH, van Schooten FJ, Bryan RT, Zeegers MP (2016). Quantified relations between exposure to tobacco smoking and bladder cancer risk: a meta-analysis of 89 observational studies. Int J Epidemiol.

[26] Stern MC, Lin J, Figueroa JD (2009). Polymorphisms in DNA repair genes, smoking, and bladder cancer risk: findings from the international consortium of bladder cancer. Cancer Res.

[27] Zhang ZF, Sarkis AS, Cordon-Cardo C (1994). Tobacco smoking, occupation, and p53 nuclear overexpression in early stage bladder cancer. Cancer Epidemiol Biomarkers Prev.

[28] Zhang ZF, Shu XM, Cordon-Cardo C (1997). Cigarette smoking and chromosome 9 alterations in bladder cancer. Cancer Epidemiol Biomarkers Prev.

[29] Chamssuddin AK, Saadat SH, Deiri K (2013). Evaluation of grade and stage in patients with bladder cancer among smokers and non-smokers. Arab J Urol.

[30] Pietzak EJ, Mucksavage P, Guzzo TJ, Malkowicz SB (2015). Heavy cigarette smoking and aggressive bladder cancer at initial presentation. Urology.

[31] Barbosa ALA, Vermeulen S, Aben KK, Grotenhuis AJ, Vrieling A, Kiemeney LA (2018). Smoking intensity and bladder cancer aggressiveness at diagnosis. PLoS ONE.

[32] Brennan P, Bogillot O, Cordier S (2000). Cigarette smoking and bladder cancer in men: a pooled analysis of 11 case-control studies. Int J Cancer.

[33] Tellini R, Mari A, Muto G (2021). Impact of smoking habit on perioperative morbidity in patients treated with radical cystectomy for urothelial bladder cancer: a systematic review and meta-analysis. Eur Urol Oncol.

[34] Piazza P, Bravi CA, Puliatti S (2022). Assessing pentafecta achievement after robot-assisted radical cystectomy and its association with surgical experience: results from a high-volume institution. Urol Oncol.

[35] Al Hussein Al Awamlh B, Shoag JE, Ravikumar V, et al (2019) Association of smoking and death from genitourinary malignancies: analysis of the national longitudinal mortality study. J Urol 202(6):1248-1254 10.1097/JU.0000000000000433 31290707

[36] Crivelli JJ, Xylinas E, Kluth LA, Rieken M, Rink M, Shariat SF (2014). Effect of smoking on outcomes of urothelial carcinoma: a systematic review of the literature. Eur Urol.

[37] Rink M, Zabor EC, Furberg H (2013). Impact of smoking and smoking cessation on outcomes in bladder cancer patients treated with radical cystectomy. Eur Urol.

[38] Cacciamani GE, Ghodoussipour S, Mari A (2020). Association between smoking exposure, neoadjuvant chemotherapy response and survival outcomes following radical cystectomy: systematic review and meta-analysis. J Urol.

[39] Wang LC, Xylinas E, Kent MT (2014). Combining smoking information and molecular markers improves prognostication in patients with urothelial carcinoma of the bladder. Urol Oncol.

[40] Koop CE, Luoto J (2006) “The health consequences of smoking: cancer,” overview of a report of the Surgeon General. 1982. Public Health Rep. 121 Suppl 1:269-275 (discussion 268) 16550790

[41] Macleod LC, Hotaling JM, Wright JL (2013). Risk factors for renal cell carcinoma in the VITAL study. J Urol.

[42] Lotan Y, Karam JA, Shariat SF (2016). Renal-cell carcinoma risk estimates based on participants in the prostate, lung, colorectal, and ovarian cancer screening trial and national lung screening trial. Urol Oncol.

[43] Gansler T, Fedewa SA, Flanders WD, Pollack LA, Siegel DA, Jemal A (2020). Prevalence of cigarette smoking among patients with different histologic types of kidney cancer. Cancer Epidemiol Biomarkers Prev.

[44] Cumberbatch MG, Rota M, Catto JW, La Vecchia C (2016). The role of tobacco smoke in bladder and kidney carcinogenesis: a comparison of exposures and meta-analysis of incidence and mortality risks. Eur Urol.

[45] Setiawan VW, Stram DO, Nomura AM, Kolonel LN, Henderson BE (2007). Risk factors for renal cell cancer: the multiethnic cohort. Am J Epidemiol.

[46] Sweeney C, Farrow DC (2000). Differential survival related to smoking among patients with renal cell carcinoma. Epidemiology.

[47] Rink M, Xylinas E, Margulis V (2013). Impact of smoking on oncologic outcomes of upper tract urothelial carcinoma after radical nephroureterectomy. Eur Urol.

[48] Rink M, Xylinas E, Trinh QD (2013). Gender-specific effect of smoking on upper tract urothelial carcinoma outcomes. BJU Int.

[49] Miyata Y, Mitsunari K, Akihiro A, Watanabe SI, Mochizuki Y, Sakai H (2015). Smoking-induced changes in cancer-related factors in patients with upper tract urothelial cancer. Mol Clin Oncol.

[50] Simonis K, Shariat SF, Rink M (2014). Urothelial Cancer Working Group of the Young Academic Urologists Working Party of the European Association of U. Smoking and smoking cessation effects on oncological outcomes in nonmuscle invasive bladder cancer. Curr Opin Urol.

